# Targeting hypoxia in solid and haematological malignancies

**DOI:** 10.1186/s13046-022-02522-y

**Published:** 2022-11-02

**Authors:** Bill Harris, Sana Saleem, Natalie Cook, Emma Searle

**Affiliations:** 1 Experimental Cancer Medicine Team, Christie NHS Foundation Trust, Manchester, UK; 2 Haematology Department, Christie NHS Foundation Trust, Manchester, UK; 3 Division of Cancer Sciences, University of Manchester, Manchester, UK

**Keywords:** Hypoxia, Cancer, Haematological, Solid tumours

## Abstract

Tumour hypoxia is a known and extensively researched phenomenon that occurs in both solid and haematological malignancies. As cancer cells proliferate, demand for oxygen can outstrip supply reducing tumour oxygenation. In solid tumours this is contributed to by disorganized blood vessel development. Tumour hypoxia is associated with resistance to treatment, more aggressive disease behaviour and an increased likelihood of metastatic progression. It can be measured using both invasive and non-invasive methods to varying degrees of accuracy. The presence of hypoxia stimulates a complex cellular network of downstream factors including Hypoxia Inducible Factor 1 (HIF1), C-X-C motif chemokine 4 (CXCR4) and Hypoxia‐inducible glycolytic enzyme hexokinase‐2 (HK2) amongst many others. They work by affecting different mechanisms including influencing angiogenesis, treatment resistance, immune surveillance and the ability to metastasize all of which contribute to a more aggressive disease pattern. Tumour hypoxia has been correlated with poorer outcomes and worse prognosis in patients. The correlation between hypoxic microenvironments and poor prognosis has led to an interest in trying to therapeutically target this phenomenon. Various methods have been used to target hypoxic microenvironments. Hypoxia-activated prodrugs (HAPs) are drugs that are only activated within hypoxic environments and these agents have been subject to investigation in several clinical trials. Drugs that target downstream factors of hypoxic environments including HIF inhibitors, mammalian target of rapamycin (mTOR) inhibitors and vascular endothelial growth factor (anti-VEGF) therapies are also in development and being used in combination in clinical trials. Despite promising pre-clinical data, clinical trials of hypoxia targeting strategies have proven challenging. Further understanding of the effect of hypoxia and related molecular mechanisms in human rather than animal models is required to guide novel therapeutic strategies and future trial design. This review will discuss the currently available methods of hypoxia targeting and assessments that may be considered in planning future clinical trials. It will also outline key trials to date in both the solid and haemato-oncology treatment spheres and discuss the limitations that may have impacted on clinical success to date.

## Background

Hypoxia is a long since recognised and widely agreed upon challenge in cancer medicine. Hypoxia in solid tumours is known to be associated with resistance to chemotherapy and radiotherapy and to promote a more aggressive tumour phenotype contributing to poor patient outcomes. The importance of hypoxia in haematological malignancy is much less studied than in the solid tumour setting, however evidence for the potential importance of bone marrow hypoxia is emerging. Interest in targeting tumour hypoxia to decrease hypoxia-associated treatment resistant mechanisms has existed for many years but has proven challenging. Several different strategies for the targeting of hypoxia have been investigated, including hypoxia activated prodrugs (HAPs) and molecular targeting of hypoxia induced resistance mechanisms. However, uncertainty remains as to the optimal methods to assess tumour hypoxia in human subjects, which contributes to a lack of understanding around which predictive and validated biomarkers of response to hypoxia targeting strategies should be used in the trial setting. Biomarkers assessing hypoxia are not routinely included in these clinical trials investigating hypoxia targeting strategies.

### Hypoxia-inducible transcription factors (HIFs)

In response to low oxygen tension, tumour cells activate gene expression programs involved in glucose uptake, metabolism, angiogenesis, proliferation, differentiation and apoptosis. The master regulators to this adaptive response are the hypoxia-inducible transcription factors (HIFs) [1]. Three isoforms of HIFα exist (HIF1α, HIF2α and HIF3α) which differ in structure and function. HIF1α is ubiquitously expressed in cells throughout the body, whereas HIF2α is expressed more abundantly during embryonic development and within vascular endothelium, lung and heart tissue. HIF3α is a repressor of HIF signalling by inhibiting the activity of HIF1/2α. For simplicity henceforth only HIF1/2α are discussed given their role in promoting tumourigenic activity and will be referred to collectively as HIFα. HIFα levels are regulated by the prolyl hydroxylase domains enzymes (PHD 1–3) which, under physiological oxygen tension, hydroxylate HIFα to allow binding of Von Hippel-Lindau (VHL; a tumour suppressor gene), ubiquitination of HIFα and subsequent proteasomal degradation. In lower oxygen tension PHD enzymes are less able to hydroxylate HIFα leading to nuclear translocation and heterodimerisation with HIFβ, and expression of hypoxia response genes via binding to hypoxia-responsive elements (HREs) in their promotor regions. It is the switching on of such gene signatures that improves survival and facilitates proliferation of tumour cells in hypoxic conditions, as well as contributing to angiogenesis, epithelial-to-mesenchymal transition (EMT), avoidance of the immune system and metastatic spread [2]. In addition, increased expression of HIFα causes up regulation of genes involved in glucose metabolism, pH regulation, cellular proliferation and apoptosis, angiogenesis and erythropoiesis [3].

### Hypoxia definitions

In its simplest terms, biochemists define hypoxia as a state is which electron transport-mediated cellular metabolism is limited by insufficient oxygen. Tissue hypoxia (be that normal or neoplastic tissue) is perhaps a better description for the phenomenon encountered in 50–60% of all solid tumours and associated with worse patient outcomes, resistance to chemotherapy and radiotherapy, and positively correlates with the extent of metastasis [4–6]. The term hypoxia is often interchangeably used with hypoxaemia which itself defines a blood oxygen partial pressure of less than 80 mmHg (10.6 kPa). Attempts to precisely define tissue hypoxia are hampered by multiple variables including metabolic demand and blood flow rate of the target tissue, arterial oxygen partial pressure, and haemoglobin concentration. It is generally accepted that the critical oxygen partial pressure, at which oxygen consumption is sufficiently reduced to alter intracellular signalling pathways, is 8—10 mmHg [7, 8]. Unregulated tumour growth, rapid cell turnover and invasion through different tissue types drive such hypoxia due to an increased oxygen demand that cannot be compensated by existing vascular access, nor oncogenic angiogenesis. Under normal circumstances hypoxia signalling pathways are activated to allow homeostasis to be achieved, often transiently, under fluctuating metabolic conditions. These signalling’survival’ pathways are hijacked during malignant transformation, the importance of which is underlined by the inclusion of metabolic reprogramming as a fundamental hallmark of cancer [9].

### Hypoxia in normal bone marrow

Unlike other normal organs that might provide the site for solid tumour development, bone marrow is considered to be physiologically hypoxic. Direct in vivo measurements of local oxygen tension (pO_2_) in the bone marrow of live mice have found intravascular pO_2_ in the range of 15–30 mmHg (mean ~ 23 mmHg, about 3% O_2_) and extravascular pO_2_ in the range of 10–25 mmHg (mean ~ 17 mmHg, about 2% O_2_) despite very high vascular density [10, 11]. Within the bone marrow architecture there are significant variations in the level of hypoxia characterised by two different niches. The endosteal niche is an area closer to the bone. The second area is a more central vascular niche which sits closer to the blood vessels. The endosteal niche is thought to be the most hypoxic area of the bone marrow and contains a higher level of HIF-1α positive cells [12, 13]. Haematopoetic stem cells (HSCs) are mostly found within the endosteal niche suggesting that there may be a role for hypoxia in the stabilisation of HSCs although this view has been challenged [10, 11].

### Hypoxia in the pathophysiology of haematological malignancies

The importance of the local hypoxic tumour microenvironment has been studied extensively in solid malignancies and there is emerging evidence to show similar detrimental hypoxia related factors are present within the bone marrow of patients with haematological malignancies.

Animal models have suggested hypoxic bone marrow in mice with multiple myeloma (MM). Comparison of the marrow of control and 5T33MM diseased mice found increased expression HIF1α suggestive of increased hypoxia in the diseased mice [14–16]. This has also been reflected in studies on human bone marrow biopsy specimens where an increased expression of HIF-1α has been demonstrated in the marrow of patients with MM [17–19]. In human subjects, circulating myeloma cells display similar characteristics with higher expression of HIF-1 found when compared to other circulating cells [20].

In acute leukaemia there are studies suggesting that the bone marrow shows a higher degree of hypoxia than in bone marrow without any malignant infiltration and that hypoxia correlates with the degree of infiltration in human and animal samples [21, 22]. Increased levels of the surrogate hypoxia markers HIF1a and Higher Vascular Endothelial Growth Factor A (VEGF-A) have been found when compared to normal bone marrow in samples from patients with Acute Myeloid Leukaemia (AML) [23]. However, HIF expression has also been demonstrated under normal oxygen tension in myeloma, leukaemia and lymphoma cells suggesting that HIF activation may act independently of hypoxia in the setting of haematological malignancy [24]. HIF has been demonstrated to play a role in the survival of cancer stem cells within the bone marrow in both leukaemia and lymphoma [24, 25].

### Hypoxia in the pathophysiology of solid tumours

Metastatic spread from tumours represents a major clinical challenge given that it is seen in more than 90% of all cancer-related deaths. Various mechanisms have been characterised by which HIF signalling drives metastatic progression in solid neoplasms. A key early aspect of metastasis is the navigation from the tissue of origin and invasion towards, usually, either the vascular or lymphatic circulation. This is associated with EMT, allowing loss of cell–cell and cell-basement membrane interactions, liberating tumour cells to invade through local structures [26]. Both induction of hypoxia and overexpression of HIF signalling in normoxia can induce EMT and promote local invasion [27, 28]. Capturing information on EMT in the clinical setting has proven challenging given the transient nature of this process and the heterogeneity within tumours, presenting both temporal and spatial obstacles to informative biopsy. There has thus been a focus on liquid biopsies, particularly involving circulating tumour cells (CTCs) to capture EMT gene signatures, although consideration must be given to changes which may occur prior to tumour cells entering the circulation. Cancer therapy affects EMT phenotype of CTCs within breast cancer patients, with those who respond to treatment having more epithelial-like CTCs compared to those with refractory disease who have more mesenchymal-like CTCs [29]. This is in keeping with pre-clinical models of breast and pancreatic cancer highlighting an important role for EMT in chemoresistance [30, 31]. This EMT transition process must be reversible (e.g. cells can transition back to epithelial phenotypes), to also allow cells to extravasate and form metastases. Whether these differences are a cause or consequence of treatment efficacy remains to be delineated.

Escaping detection and targeting by the immune system is key to survival as tumours extend and invade from their tissue of origin. Immunotherapy in the form of checkpoint inhibitors has been an important development in solid oncology in the last decade and remains a key focus for drug development. HIF signalling impacts directly on several key immune cell types, all of which act to promote an immunosuppressive microenvironment [32–35]. T cell receptor (TCR) signal transduction is negatively regulated by HIF1α inhibiting effector T cell-mediated tumour cell targeting [36]. Experiments in both prostate and breast cancer cell lines revealed increased Programmed death-ligand 1 (PD-L1) expression via increased HIF1α expression and subsequent binding at HREs in the PD-L1 promoter in hypoxic (0.5% O2) versus normoxic (20% O2) conditions. This conferred significantly reduced cytotoxic T cell lysis in both a PD-L1 and HIF1α-dependent manner, likely due to interaction with Programmed cell death protein 1 (PD-1) on effector T cells to escape immune detection [37, 38].

In hypoxic conditions, mismatch repair capacity is reduced and leads to a greater level of microsatellite instability. This is in part controlled by HIF signalling at both the transcriptional and translational level, likely as a conserved physiological adaptation to diminished metabolic resource [39, 40]. Inhibition of complimentary repair mechanisms such as with protein poly(ADP-ribose) polymerase-1 (PARP-1) has been hypothesised to generate a synthetic lethal interaction in hypoxic tumour cells [41]. Two phase I clinical trials have investigated the combination of PARP inhibition together with angiogenesis inhibition. The first combined the PARP inhibitor Olaparib with the VEGF inhibitor Bevacizumab in twelve heavily pre-treated patients with advanced refractory solid tumours. Unfortunately, nine of the twelve discontinued due to either disease progression or toxicity to treatment [42]. The second study combined Olaparib with a novel VEGF inhibitor cediranib in 28 patients with either recurrent epithelial ovarian or triple-negative breast cancer. Some putative evidence of efficacy was seen in ovarian cancer patients but 75% of patients discontinued the study due to ≥ grade 3 toxicities [43]. Neither study progressed to later phases of development.

### Angiogenesis and hypoxia

Angiogenesis has long been noted to play a role in the pathogenesis and progression of various different types of cancer, with HIF signalling implicated in regulating the process directly for almost as long. The presence of HRE within the VEGF promotor region confirms a direct link but, further, HIF signalling directly or indirectly regulates more than 2% of all genes associated with neovascularisation [44]. Several clinical studies have looked to address this causal relationship via combination therapy in anticancer therapy. Bevacizumab is an established VEGF inhibitor whilst Temsirolimus inhibits mTOR, an upstream regulator of HIF signalling through the PI3K/AKT/mTOR pathway. A phase I clinical trial of these agents in combination with liposomal doxorubicin revealed a 19% objective response rate in 74 breast and gynaecological cancer patients [45]. A larger study using Bevacizumab, Temsirolimus and Sorafenib, an inhibitor of multiple kinases involved in cell proliferation and angiogenesis, found no progression-free survival benefit compared with Bevacizumab monotherapy in 331 advanced renal cell carcinoma patients [46]. A further phase I study combined Bevacizumab with Bortezomib, a proteosome inhibitor which indirectly inhibits HIF signalling through Phosphoinositide 3-kinase (PI3K)/Akt/mTOR deactivation, in 91 patients with advanced refractory solid malignancies. Disappointingly, only 12% of these patients had either an objective response or stable disease at six months [47] and this regimen has not progressed to later phase trials. Importantly, these studies did not include the prospective use of any validated hypoxia or VEGF pathway assays or biomarkers so mechanisms of resistance remain unknown.

Within non-Hodgkin’s lymphoma (NHL), a recent study has compared malignant lymph node biopsies from diagnosis and at recurrence of disease. Reactive lymphadenopathy archival biopsies were analysed as a negative control. The lymphoma cells within lymph node biopsies reviewed at the point of NHL recurrence showed a significantly increased vascular network and higher level of HIF-1a expression suggesting a correlation between angiogenesis, hypoxia and disease progression [48].

Similarly, it has been shown in patients with multiple myeloma that HIF1a and HIF2a were strongly expressed within the myeloma cells alongside an up-regulation of VEGFR. This up-regulation was linked to increased angiogenesis. This was linked to a worse prognosis in MM cases that showed a high vascular density [18]. It is speculated that many of the traditional cytotoxic therapies used to treat patients with multiple myeloma may exert some of their effects through reducing expression of HIF1 (and in turn VEGF) thereby suppressing neo-angiogenesis [49, 50].

### Treatment resistance

Evidence exists that hypoxic tumour microenvironments can interfere with the efficacy of traditional chemotherapy agents on tumour activity in both solid and haematological malignancies. It has been shown that hypoxia of bone marrow can lead to arrest of the cell cycle of AML blasts in the G0/G1 phase therefore not reaching the S phase. Cytarabine, a conventional chemotherapy which is the mainstay of much AML treatment, is an S phase dependent drug. When Cytarabine was applied to AML blasts exposed to hypoxic conditions it was shown to have a significantly decreased affect [51]. Hypoxia associated treatment resistance has also been demonstrated in Acute Lymphoblastic Leukaemia (ALL), a study found that blocking HIF1a expression resulted in increased sensitivity to cytotoxic therapy [52].

Several studies within a multiple myeloma population have shown that inhibition of downstream enzymes in the hypoxia pathway can increase susceptibility to cytotoxic therapy. Ikeda et al. demonstrated that exposure to an antibody against hypoxia‐inducible glycolytic enzyme hexokinase‐2 (HK2) increased apoptosis [53]. HK2 has also been found to contribute to an anti-apoptotic effect in myeloma cells whilst in vivo studies have found increased efficacy of the chemotherapy agent melphalan in the presence of an inhibitor of HIF1α [54, 55]. The mechanism by which HIF signalling inhibition sensitises to melphalan therapy remains to be elucidated.

Increased HIF1α expression significantly and inversely correlated with response to Epirubicin therapy [56] and was also shown to be an independent risk factor for resistance to aromatase inhibitor therapy [57] in 187 and 114 oestrogen receptor (ER) positive breast cancer patients, respectively. Histone deacetylation has been shown to stabilise HIF1α as acetylation leads to polyubiquitination and targeting toward proteosomal degradation [58]. Interestingly, histone deacetylase inhibition (HDACi) reduces HIF1α expression through a VHL-independent mechanism [59]. Preclinical work has revealed HDACi can reverse treatment resistance in combination with the VEGF inhibitor pazopanib in sarcoma cell lines [60]. In a phase I trial the HDAC inhibitor Abexinostat was used in combination with Pazopanib in 51 patients with advanced renal cell carcinoma. Tumour regression was seen in seven of 10 patients with previously pazopanib-refractory disease indicating a potential role for HIF signalling in VEGF treatment resistance clinically [61].

HIFα plays a role in chemotherapy resistance through the activation of the multidrug resistance 1 (MDR1) gene in hypoxic conditions. A seven-fold increase in MDR1 was seen via quantitative microarray in epithelial cells exposed to hypoxia [62]. In human lung adenocarcinoma cells under hypoxic stress HIFα and multidrug resistance levels were increased, as was resistance to Adriamycin [63]. Clinically MDR1 is expressed more highly in triple negative breast cancer (TNBC) compared to other breast cancer subtypes which correlates with greater chemoresistance and poorer prognoses [64, 65]. Despite the mounting evidence for targeting multidrug resistance in cancer clinically there has been limited success in either solid or haematological cancers to date [66, 67]. Most of the assessments investigating resistance have been performed retrospectively and examined at the end of trials rather than incorporating prospective biomarkers to understand mechanisms at the outset.

## Clinical assessment of hypoxia

There are multiple different methods used for the assessment of hypoxia, each has its advantages and disadvantages, and these are summarised in Table 1. In broad terms these can be broken down into direct methods, tissue-based methods and imaging techniques. The majority of these assessment methods have been investigated in solid malignancies and little evidence is currently available for these methods in haematological malignancies.

**Table 1:** Advantages and disadvantages of the methods of assessing hypoxia

Method	Advantages	Disadvantages
Oxygen Electrodes	Around 100 measurements taken- good overview of area No major adverse effects	Surface lesions only Invasive No repeat measurements Cannot account for necrotic areas- will give discordant results Artifacts-excessive oxygen consumption Technically skilled user and inter-operator variability
Phosphorescence quenching	Real time oxygenation information Readings are independent of tracer concentrations	Invasive technique Technically skilled user Early in development- limited availability
Electron Paramagnetic Resonance oximetry	Implantable technology- repeated results. Can monitor effects of treatment Absolute pO_2_ readings	Invasive- needs a direct probe in situ Early in development- limited availability
Endogenous markers	Not affected by the sampling time or microenvironment It can be correlated within the same sample against other markers of tumour hypoxia	Cell line specific Can be affected by metabolic factors that vary between cells
Dynamic contrast-enhanced magnetic resonance imaging	Non-invasive Widely available Radiology departments familiar with method and equipped to perform and report imaging Can be repeated to monitor effects of treatment with relative accuracy	When administered IV mostly absorbed in liver/spleen. Amount in tumours often insufficient to get an accurate reading Cleared within days- limited time period for collecting data When administered into tumour can only read oxygen tension within that area of the tumour Readings significantly affected by temperature
Blood-oxygen level dependent magnetic resonance imaging	Non-invasive Can detect changes in tumour hypoxia over time	Small movements can lead to poor images and artefact Not a direct measure of oxygenation and therefore independent variables can interfere with measurements
Positron emission tomography imaging	Non-invasive Widely available Familiar method- clinicians and radiology departments used to dealing with images and results Repeated measurements possible Enables the visualization of the hypoxic status of the entire tumour in 3D image	Varying tracers used result in varying uptake levels and result in some discrimination between hypoxic levels Relatively short half life of tracer means it must be manufactured and imaged within several hours
Pimonidazole	Non-invasive Good prognostic correlation	Limited availability Requires tumour biopsy after administration of Pimonidazole- tumour needs to be accessible Invasive

### Direct methods

Long considered the gold standard method by many researchers in the field, oxygen electrodes are one of the oldest and most studied methods of direct measurement of hypoxia. The electrodes are polarographic needles inserted directly into a tumour or metastatic lymph node with the purpose of measuring oxygen partial pressure (pO2). They rely on the interaction of oxygen with a sensor on the probe and the method is based on the electro-reduction of oxygen molecules. The sensors measure oxygen at various points along their length and therefore can provide a good overall view of the oxygen levels of the tumour. However, some concern exists as to whether the oxygen electrodes could contribute to seeding of the tumour [68]. There are barriers to the utilisation of oxygen electrodes within the context of a clinical trial, including lack of availability of probes, skilled probe operators, patient acceptability and a reliance on an assessable tumour location. Oxygen electrodes are also particularly ill-suited as a method of hypoxia assessment in haematological malignancies where tumour cells may be predominantly confined within the bone marrow.

### Tissue based methods

Tissue based methods of hypoxia assessment all require the removal of a sample of tumour tissue. These are necessarily invasive and reliant on the accessibility of the tumour. However, these methods do allow for the centralisation of hypoxia assessment as part of a clinical trial. Pimonidazole is a 2-nitroimidazole compound which undergoes a nitro reductase catalysed single-electron reduction in the presence of hypoxia. Pimonidazole then binds covalently to cellular compartments in hypoxic cells [69] and can then be detected in poorly oxygenated regions in histological sections from tumours [70]. Pimonidazole has been used to detect hypoxic areas within solid tumours [71–73] and the marrow of AML patients’ populations [74]. Pimonidazole can be safely administered to patients in oral and intravenous forms and after removal of a tumour sample, binding of pimonidazole can be assessed histologically in tumour sections or by flow cytometry using anti-pimonidazole antibodies. As a method of assessing tumour hypoxia, pimonidazole can be considered to give an average of hypoxia during the period of pimonidazole metabolism.

### Endogenous markers

There are several endogenous markers that have a role in the assessment of hypoxia within both solid and haematological malignancies. Histological assessment of the levels of these surrogate hypoxia markers is possible using primary antibodies targeted against these proteins. Carbonic anhydrase (CA) is an enzyme that catalyses the reversible hydration of carbon dioxide to carbonic acid. Carbonic anhydrase 9 (CAIX) is strongly induced by hypoxia and has been implicated in hydrogen ion efflux and prevention of cell death in hypoxia [75]. Whilst CAIX has shown some promise of correlation with prognosis in several solid tumours [76, 77], it did not correlate well with other measurements of hypoxia (pimonidazole staining and direct p0_**2**_ measurements) [78, 79].

Glucose transporter 1 (GLUT-1) is a membrane protein involved in transporting glucose across cell membranes. During hypoxic conditions there is an increased rate of glycolysis and therefore this transporter is up regulated in order to facilitate the increased glucose requirements.

Osteopontin (OPN) is a tumour associated phosphorylated glycoprotein. It is found is a variety of different cells and plays a role in modulating cell adhesion and in angiogenesis. It is known to be upregulated in hypoxic environments [80]. There are several studies which show that osteopontin may act as a surrogate hypoxia marker and therefore as a marker of prognosis in various cancer patient populations [81, 82].

### Imaging techniques

Overhauser-enhanced magnetic resonance imaging (OMRI) is essentially a combination of MRI and electron paramagnetic resonance (EPR) methods of assessing for hypoxia which allows for anatomical tissue imaging alongside physiological parameter measurements. Essentially novel contrast medium based on single electron substance allows single enhancement which is influenced by oxygen concentration via low-field MRI scanning. To date this has only been explored in the preclinical setting but offers promise for an accurate measure of tissue hypoxia in cancer patients [83].

Dynamic contrast enhanced MRI can be used to look at perfusion data which in turn can estimate tissue oxygen tension. In both preclinical and initial clinical studies this method has shown a great deal of promise in being able to identify poorly perfused and hypoxic areas of tumour [84, 85]. This is yet to be used prospectively in combination with hypoxia targeting agents.

Blood-oxygen level dependent MRI (BOLD MRI) is a technique used within functional MRI studies which works by relying on the differences in blood flow to determine regional oxygen levels and identify hypoxia. It has been demonstrated to have a high sensitivity to hypoxic regions in the tumours of patients with prostate cancer when compared with both Pimonidazole staining and oxygen electrode readings [71, 86]. Additionally, BOLD-MRI has been shown to reliably yield hypoxic information in patients with breast and cervical cancer [87–89].

Positron emission tomography (PET) imaging is a non-invasive technique which uses radioisotopes to determine the presence of tumour hypoxia. The tracers are given intravenously and the uptake into tissues is caught by using a PET camera. In hypoxic conditions, the tracer is chemically reduced and bonds with thiol-rich proteins and this compound accumulates intracellularly. It has been shown to produce reliable results within cervical cancer and head and neck cancers [90, 91].

## Hypoxia targeting strategies

Given that tumour tissues are differentially more hypoxic than their wild type counterparts and hypoxia is associated with increased chemoradiotherapy resistance, hypoxia targeting strategies have been extensively researched in both the pre-clinical and clinical settings. The strategies can be broadly categorised into hypoxia activated prodrugs (HAPs) and drugs that act either up- or down-stream of the HIFα signalling pathway.

### Hypoxia-activated Prodrugs (HAPs)

Also known as bio reductive agents, these compounds are selectively reduced under hypoxic conditions to produce activated cytotoxic drugs so have relatively little toxicity to normoxic tissue. The HAPS most extensively investigated in both the preclinical and clinical setting include Tirapazamine (SR-4233), Apaziquone (EO9), PR-104, Banoxantrone (AQ4N) and Evofosfamide (TH-302). They largely exert their cytotoxic effect by interfering with normal DNA replication and, further, tumour cell division and proliferation [92–95]. Evofosfamide is a second-generation HAP and has been of particular interest in recent years. It consists of a dual moiety of bromo-iso-phosphoramide (Br-IPM), a DNA cross-linking mustard prodrug, and 2-nitroimidazole, a bioreductive phosporamide prodrug. Both undergo reduction reactions to activate the prodrugs in hypoxic conditions [96].

### HIF Pathway Inhibitors

Pharmacological targeting of the HIF signalling pathway is complicated by its interconnected interactions and redundancy with other signalling pathways. In the last two decades many and varied targeting strategies have been developed including inhibitors of HIF1α transcription, translation, protein stabilisation and heterodimerisation with HIFβ. HIF signalling may be targeted indirectly either through the upstream PI3K/Akt/mTOR pathway (such as the mTOR inhibitors temsirolimus, evorolimus and sirolimus), or downstream, through anti-VEGF therapy (such as Bevacizumab or multiple kinase inhibitors like Lenvatinib and Sorafenib which inhibit VEGFR 1/2/3 alongside fibroblast growth factor receptors (FGFR) 1/2/3/4, Platelet-derived growth factor receptor (PDGFR), c-KIT and the RET oncogene). A detailed review of these different targeting strategies is beyond the scope of this article and has been covered elsewhere [97].

Of interest, given that hydroxylation via PHD proteins plays such a pivotal role in reducing HIFα levels via von Hippel-Lindau (VHL) mediated proteosomal degradation, benzopyranyl 1,2,3-triazole has recently been identified as a novel anticancer agent. This compound increases HIFα hydroxylation and thus subsequent targeting for proteosomal degradation, reduces VEGF expression and angiogenesis in both in vitro and in vivo cancer models as well as showing combination efficacy with the epidermal growth factor receptor (EGFR) receptor gefitinib [98].

Another potentially druggable HIF-related target is Heat shock protein 90 (HSP90) which binds to and stabilises HIFα to increase its activity by; (i) blocking VHL-dependent proteosomal degradation, and (ii) improving HIF heterodimer recruitment of further transcriptional machinery at HREs [99]. Multiple HSP90 inhibitors including Geldanamycin semi-synthetic derivatives such as tanespimycin and farnesyltransferase derivatives have been shown to reduce HIFα levels and downregulate HRE-containing downstream genes in human cancer settings [100, 101]. In a phase I trial, tanespimycin was used in combination with bortezomib for 17 patients with advanced refractory solid tumours but unfortunately no objective responses were seen [102].

Camptothecins (CPTs), including Topotecan, which was originally discovered as part of a HIF-targeted transcriptional activity assay [103]. They are traditional chemotherapeutic agents which act as topoisomerase I inhibitors but also prevent HIF1α accumulation and have been shown to reduce hypoxia-mediated VEGF mRNA expression in human glioma cell lines under hypoxic conditions [104]. Recently CRLX-101 was developed as a first-in-class nano pharmaceutical agent which conjugates a CPT moiety to a polyethene glycol (PEG) co-polymer [105] and has shown higher efficacy and improved tolerability compared with synthetic analogues Topotecan and Irinotecan [106]. It has shown anticancer efficacy in combination with Bevacizumab in triple negative breast cancer mouse models [107] as well as monotherapy for locally advanced rectal cancer [108]. Two phase II clinical trials have explored CRLX-101 in combination with Bevacizumab to treat advanced renal cell carcinoma although sadly neither displayed any improved anticancer efficacy compared with approved treatment agents [109, 110]. Most trials discussed above have not investigated mechanisms of resistance or reasons behind the disappointing efficacy results. There was also limited use of prospective pharmacodynamic biomarkers assessing baseline hypoxia, or changes in hypoxia levels in patients on the trials.

## Clinical trials of hypoxia targeting strategies

The most significant advance in HIF pathway targeting strategies came in recent months with The United States Food and Drug Administration (FDA) approval of the HIF2α inhibitor Belzutifan for the treatment of von Hippel-Lindau associated tumours including renal cell carcinomas, central nervous system haemangiomas and pancreatic neuroendocrine tumours. This follows the publication of phase II clinical trial findings by Jonasch et al. [111]. This study recruited patients with renal cell carcinoma secondary to von Hippel-Lindau syndrome and used objective response (including complete and partial responses) as the primary endpoint. Objective response was seen in 49% of patients with renal cell carcinoma, in 77% of co-existing pancreatic neuroendocrine tumours and in 30% of co-existing central nervous system haemangiomas. 100% of co-existing retinal haemangiomas (16 eyes across 12 patients) were graded as showing some improvement following Belzutifan administration. This work follows on from several phase I trials which screened novel HIF2α inhibitors in von Hippel-Lindau associated tumours [112, 113]. A summary of these studies along with recent clinical trials utilising hypoxia-targeting strategies is summarised in Table 2 [102, 109–134].

**Table 2:** Summary of published clinical trials using hypoxia targeting strategies

Target	IMP	Treatment	Trial Phase	Patients Treated	Disease type	Findings	Reference
**Hypoxia-activated Prodru**gs	Evofosfamide (TH-302)	Pazopanib + Evofosfamide	I	30	All solid tumours	Partial response in 10%, stable disease in 57%, progressive disease in 23% of patients	(Riedel et al., 2017) [114]
		Evofosfamide monotherapy in relapsed/refractory leukaemia	I	49	Acute myeloid/lymphoid leukaemia	Reduced HIF1a/CAIX but only 6% overall response rate	(Badar et al., 2016) [115]
		Gemcitabine Vs Gemcitabine + Evofosfamide	II	214	Pancreatic	Extended progression-free survival (5.6 vs 3.6 months; *p* = 0.005), greater reduction in tumour burden (*p* = 0.04) and CA19.9 levels (*p* = 0.008) with addition of Evofosfamide. No significant difference in overall survival	(Borad et al., 2015) [116]
		Evofosfamide + Dexamethasone ± Bortezomib	I-II	59	Multiple myeloma	Stable disease (38/59) or better in 80% patients across all cohorts	(Laubach et al., 2019)
		Doxorubicin Vs Doxorubicin + Evofosfamide	III	640	Soft-tissue sarcoma	No survival benefit (18.4 months combination therapy Vs 19.0 months Doxorubicin monotherapy median overall survival)	(Tap et al., 2017) [117]
		Gemcitabine Vs Gemcitabine + Evofosfamide	III	693	Pancreatic	Overall survival endpoint not quite met (8.7 months combination therapy Vs 7.6 months Gemcitabine monotherapy; *p* = 0.059). Median progression-free survival 5.5 months combination therapy V 3.7 months Gemcitabine monotherapy (*P* = 0.004)	(Van Cutsem et al., 2016) [119]
	Tirapazamine (SR-4233)	Tirapazamine (TPZ) + Carboplatin + Paclitaxel	I	42	All solid tumours	8% complete response, 5% partial response, 60% stable disease, 26% progression of disease	(Lara et al., 2003) [120]
		Cisplatin + radiotherapy + Tirapazamine	I	16	Oesophageal adenocarcinoma	Three year overall survival 88%, but omission of Tirapazamine needed in latter cycles to avoid dose-limiting toxicity of neutropenia	(Rischin et al., 2001) [121]
		Arterial Embolisation + Tirapazamine	I	27	Hepatocellular carcinoma	60% complete response, 84% objective response	(Abi-Jaoudeh et al., 2021) [122]
		Cisplatin + Etoposide + radiotherapy + Tirapazamine	II	69	Limited-stage small cell lung cancer	Median progression-free survival 11 months, median overall survival 21 months	(Le et al., 2009) [123]
		Paclitaxel + Carboplatin ± Tirapazamine	III	367	Non-small cell lung cancer	Overall survival end-points not reached, significantly more adverse events leading to treatment cessation when Tirapazamine added to combination therapy (p < 0.05), mostly due to myelosuppression	(Williamson et al., 2005) [124]
	PR-104	PR-104 + Docetaxel or Gemcitabine	I	42	All solid tumours	9.5% partial response overall, significant myelosuppression prevented further analysis of combo + Gemcitabine	(McKeage et al., 2012) [125]
		PR-104	I	27	All solid tumours	No objective responses were observed	(Jameson et al., 2010) [126]
		PR-104	I-II	50	Acute myeloid/lymphoid leukaemia	Objective response in 32% AML and 20% ALL patients	(Konopleva et al., 2015) [127]
**HIF Signalling**	Belzutifan	Belzutifan	I	98	Renal cell carcinoma	Objective response in 25%, median progression-free survival was 14.5 months	(Choueiri et al., 2021) [112]
		Belzutifan	II		VHL-associated tumours	Objective response in 49% renal cell carcinomas, 77% pancreatic lesions, 30% CNS haemangioblastomas, 100% retinal haemangioblastomas	(Jonasch et al., 2021) [111]
	PT2385	PT2385	I	51	Renal cell carcinoma	2% complete response, 12% partial response, 52% stable disease	(Courtney et al., 2018) [113]
	CRLX101	CRLX101 + Bevacizumab	I-II	22	Renal cell carcinoma	23% partial response, 55% achieving progression-free survival of more than four months	(Keefe et al., 2016) [109]
		CRLX101 + Bevacizumab Vs standard of care (SOC) therapy	II	111	Renal cell carcinoma	No improvement in progression-free survival (3.7 months CRLX101 + Bevacizumab Vs 3.9 months SOC therapy; *p* = 0.831) or objective response (5% CRLX101 + Bevacizumab Vs 14% SOC therapy; *p* = 0.836)	(Voss et al., 2017) [110]
	PX-12	PX-12 (thioredoxin-1 inhibitor)	I	38	All solid tumours	18% stable disease, as best response observed	(Ramanathan et al., 2007) [128]
		PX-12	I	14	All solid tumours	7% stable disease, as best response observed	(Ramanathan et al., 2012) [129]
	Tanespimycin	Tanespimycin + Bortezomib	I	17	All solid tumours	6% stable disease, as best response observed	(Schenk et al., 2013) [102]
**CXCR4 (haematological malignancies)**	BL-8040	BL-8040 + Ara-C	II	42	Acute myeloid leukaemia	29% complete remission ± incomplete haematological recovery. Median overall survival 8.4 months	(Borthakur et al., 2021) [130]
	Plerixafor	Plerixafor + high-dose cytarabine + etoposide	I	19	Acute myeloid/lymphoid leukaemia, myelodysplastic syndrome	16% objective response, exclusively in acute myeloid leukaemia	(Cooper et al., 2017) [131]
		Plerixafor + Decitabine	I	69	Acute myeloid/lymphoid leukaemia, myelodysplastic syndrome	43% objective response	(Roboz et al., 2018) [132]
		Plerixafor + FLAG-IDA	I-II	41	Acute myeloid leukaemia	Complete remission ± incomplete haematological recovery in 50% and 47% of primary refractory and early relapse groups respectively	(Martínez-Cuadrón et al., 2018) [133]
	Ulocuplumab	Ulocuplumab + MEC (mitoxantrone + etoposide + cytarabine)	I	73	Acute myeloid leukaemia	Complete remission ± incomplete haematological recovery in 51% combination therapy compared with 24–28% in those receiving MEC alone	(Becker et al., 2014) [134]

*Evofosfamide* is a second-generation hypoxia-activated prodrug (HAP) consisting of a dual moiety in which bromo-iso-phosphoramide (Br-IPM) is attached to the enzyme responsible for its reduction-dependent activation, 2-nitroimidazole. *Tirapazamine* generates an oxidative radical following reduction in hypoxic conditions. This occurs preferentially in the nucleus leading to DNA double-strand breaks, chromosomal degradation and ultimately to apoptosis. *PR-104* contains a nitrogen mustard moiety which, when activated by reduction in hypoxia, is able to cross-link DNA to prevent further replication. *Belzutifan* is a small molecule selective HIF2α inhibitor. *PT2385* similarly acts as an antagonist of HIF2α. *CRLX-101* is a nanopharmaceutical agent which conjugates a camptothecin moiety to a polyethene glycol co-polymer. PX-12 is a small molecule inhibitor of thioredoxin-1 (Trx-1), a redox protein pivotal for HIF1α and VEGF. Tanespimycin is a Geldanamycin semi-synthetic derivative inhibitor of heat shock protein 90 (HSP90) which binds to and stabilises HIF1α. *BL-8040* is a CXCR4 antagonist, a downstream target of HIF1a. *Plerixafor* is similarly a CXCR4 antagonist whilst *Ulocuplumab* is a fully human IgG4 monoclonal antibody which prevents the binding of CXCR4 to CXCL12

Published clinical literature exists regarding hypoxia-related biomarker analyses to help identify potential markers with therapeutic prognostic value. MicroRNA-210 (miR-210) is upregulated in tissue hypoxia [135] and has been linked to improved tumour cell survival and impaired DNA repair [136, 137]. Ono and colleagues accessed plasma samples from melanoma patients enrolled on a phase III trial and analysed circulating cell-free miR-210 via quantitative Polymerase chain reaction (PCR). They found miR-210 to be significantly higher in metastatic versus primary disease and a significant positive correlation with poorer prognosis (*p* < 0.001). Interestingly, when analysing sequential serum samples from individual patients miR-210 levels significantly increased in the three-month period prior to disease recurrence (*p* = 0.012) [138]. Irlam-Jones and colleagues found that miR-210 level significantly and positively correlated with hypoxia signalling, including HIF1α (*p* = 0.01) and carbonic anhydrase 9 (CAIX) level (*p* = 0.0004) as well as a 26-gene hypoxia score (*p* = 0.07), but concluded this did not improve on these established hypoxia biomarkers [139].

CAIX is downstream of and dependent upon HIF signalling. As a metalloenzyme, CAIX catalyses the production of H^+^ and HCO3^−^ from H_2_O and CO_2_ which helps to buffer pH fluctuations in hypoxic tumour microenvironments [140]. Higher CAIX expression was significantly associated with poorer survival outcomes (*p* = 0.016) in 45 glioblastoma multiforme and anaplastic astrocytoma patients treated with bevacizumab and irinotecan in a phase II clinical trial [141]. Similarly, higher CAIX expression was negatively correlated with two-year loco-regional control (*p* = 0.001) in 39 head-and-neck squamous cell carcinoma (HNSCC) patients receiving chemoradiation in a prospective imaging trial [142]. A larger cohort of 203 soft tissue sarcoma patients were analysed via immunohistochemistry retrospectively for the hypoxia markers HIF1α, GLUT1 and CAIX following a phase III radiotherapy trial. Whilst HIF1α and GLUT-1 protein expression were not prognostic, high CAIX expression was significantly associated with worse disease-free survival outcomes (*p* < 0.001) indicating that this downstream factor in HIF signalling may be a more clinically significant prognostication marker [143]. However it is as yet unclear whether this is due to a functional role of CAIX, i.e. in hydrogen ion efflux to promote cancer cell survival, or a result of differential protein stability or staining techniques utilised in this study.

## Conclusion

The role of hypoxia in cancer is not in doubt. Hypoxia has been consistently shown to contribute to more aggressive and treatment resistant disease in both solid and haematological malignancies. Hypoxia modulates the growth and characteristics of cancer via an array of highly complex pathways as summarized above, but the many ways in which hypoxia is important in cancer remains an expanding area of research.

The ability to identify hypoxia, measure it with precision and work out in which patients it is especially important, is essential to further progress with hypoxia targeting strategies in the clinical setting. To date, no large studies identifying hypoxia in specific cancer patient populations have been performed. The failure to accurately identify patients with hypoxic tumours, and the lack of integration of validated hypoxia biomarkers into clinical trials, has contributed to disappointing clinical trial results. Whilst the gold standard for measuring hypoxia is currently considered to be oxygen electrodes, there are obvious benefits to using imaging modalities, being non-invasive and independent of operator differences. The development of hypoxia biomarkers provides future promise for alternative effective tools to identify patients who may benefit from clinical trials of hypoxia targeting strategies. However, data is still limited to a handful of tumour types in solid tumours and none currently exists in the haematological malignancy setting. Perhaps one of the biggest flaws to date in clinical trials targeting hypoxia pathways in cancer has been a failure to first pre-screen patients based on established and validated hypoxia biomarkers, and then only enrol those patients with proven hypoxic tumours onto trials of hypoxia targeted agents. Ultimately a panel of biomarkers will probably be needed as we anticipate different hypoxia markers are likely to prove prognostic in different cancer types. Future clinical trials also need to include pharmacodynamic biomarkers of hypoxia so we can also further understand mechanisms of response and resistance to hypoxia targeting strategies.

**Fig. 1:**
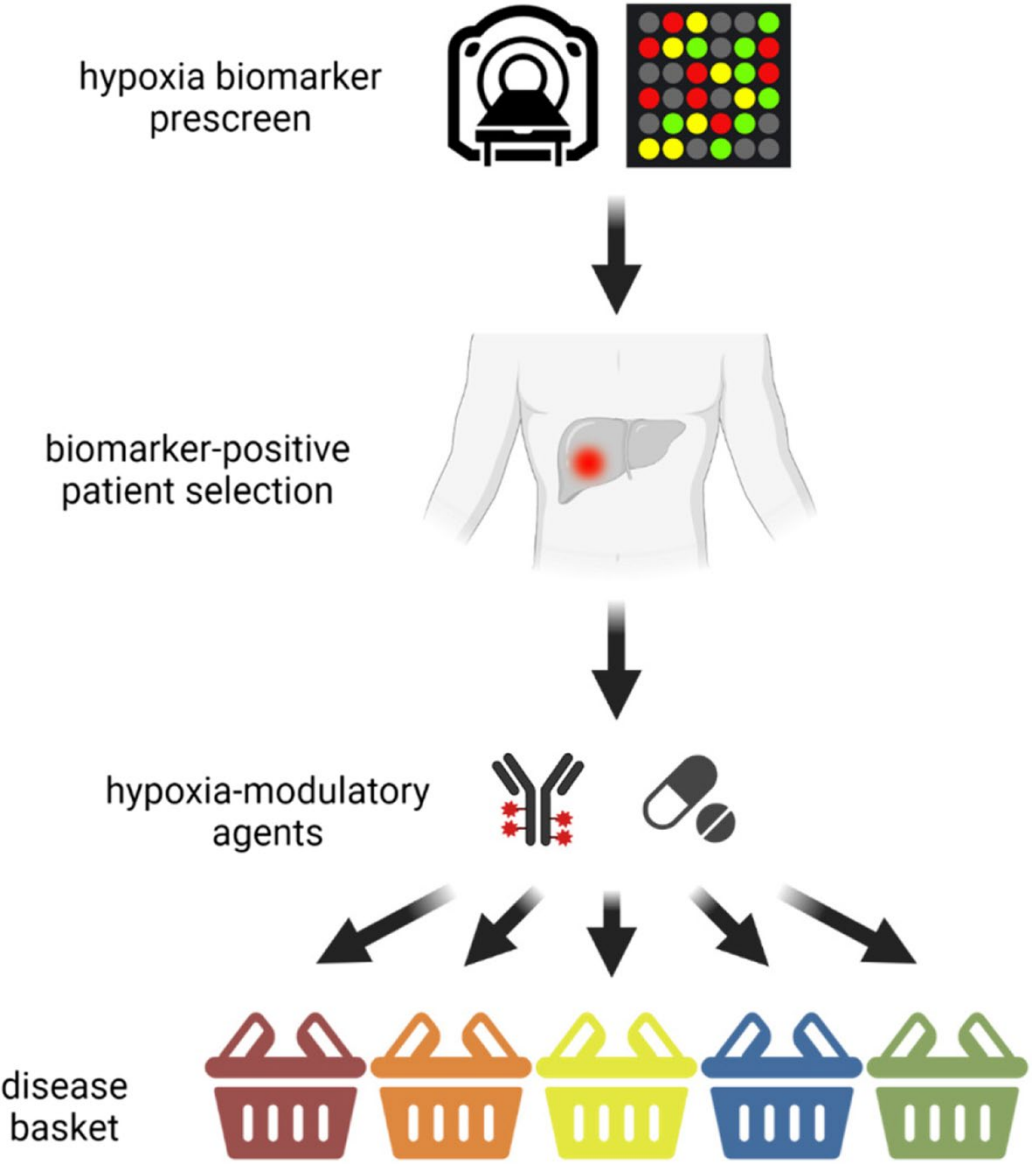
Suggested future clinical trial algorithm for hypoxia biomarker integration. Patients consent for pre-screening to investigate hypoxia biomarker expression in their cancer (detected either via hypoxia based imaging (i.e. pimonidazole) or validated hypoxia gene assays). Only patients that test positive for the hypoxia biomarker can then consent to the main study of a hypoxia modulating agent. Patients are stratified into disease specific cohorts based on the cancer type of interest (e.g. sarcoma, bladder, pancreas) due to the differences in cancer specific outcomes in these cohorts. If any hypoxia-biomarker cohort in this basket design shows sufficient signal of clinically relevant activity (e.g. durable clinical benefit) to warrant further investigation the cohort can then be expanded

Figure 1 is an example of how future clinical trials could be designed to propel forward knowledge and experience in this area of cancer research. Performing initial pre-screening assessments using validated hypoxia biomarkers has potential to identify the population of patients where hypoxia is contributing to disease progression. Once this population of patients has been identified their treatment could be supplemented with hypoxia-modulatory agents and outcomes monitored. The results from these trials would allow us to assess for clinically relevant activity and take forward any of the promising agents to further larger later phase clinical trials.

## Data Availability

Not applicable.

## Abbreviations

ALL: Acute lymphoblastic leukaemia
AML: Acute myeloid leukaemia
BOLD MRI: Blood-oxygen level dependent MRI
Br-IPM: Bromo-iso-phosphoramide
CA: Carbonic anhydrase
CAIX: Carbonic anhydrase 9
CMML: Chronic myelomonocytic leukaemia
CPT: Camptothecin
CTC: Circulating tumour cell
CXCR4: C-X-C motif chemokine 4
EGFR: Epidermal growth factor receptor
EMT: Epithelial-to-mesenchymal transition
EPR: Electron paramagnetic resonance
ER: Oestrogen receptor
FDA: The United States Food and Drug Administration
FGFR: Fibroblast growth factor receptors
GLUT-1: Glucose transporter 1
HAPs: Hypoxia-activated prodrugs
HDAC: Histone deacetylase
HIF: Hypoxia Inducible Factor
HK2: Hypoxia‐inducible glycolytic enzyme hexokinase‐2
HNSCC: Head-and-neck squamous cell carcinoma
HRE: Hypoxia-responsive elements
HSC: Haematopoetic stem cells
HSP90: Heat shock protein 90
NHL: Non-Hodgkin’s lymphoma
MDR1: Multidrug resistance 1
MHC: Major histocompatibility complex
MM: Multiple myeloma
mTOR: Mammalian target of rapamycin
OPN: Osteopontin
OMRI: Over Hauser-enhanced magnetic resonance imaging
PARP: Protein poly(ADP-ribose) polymerase
PCR: Polymerase chain reaction
PEG: Polyethene glycol
PDGFR: Platelet-derived growth factor receptor
PD-L1: Programmed death-ligand 1
PD-1: Programmed cell death protein 1
PET: Positron emission tomography
PHD: Propyl hydroxylase domains enzymes
PI3K: Phosphoinositide 3-kinase
TCR: T cell receptor
TNBC: Triple negative breast cancer
VEGF: Vascular endothelial growth factor
VHL: Von Hippel-Lindau
